# Arteriosclerosis

**Published:** 1913-06

**Authors:** 


ARTERIOSCLEROSIS. By James Third, M. D., *Can. Pract. and Rev., April,* 1913. Bearing on this question the following statistics are cited from Ontario reports:

| | Total Deaths For Year. | Deaths, 50-60. | 70 and Up. |
|---|---|---|---|
| Clergymen ....... | 61 | 11.5% | 44.3% |
| Barristers ....... | 33 | 12 % | 33.3% |
| Physicians ....... | 60 | 33.3% | 21 % |
| Carpenters ....... | 308 | 18 % | 31 % |
| Blacksmiths ...... | 112 | 18 % | 40 % |
| Farmers ......... | 3,229 | 12 % | 50 % |

